# Laparoscopy-assisted distal gastrectomy for early gastric cancer poses few limitations for selected elderly patients: a single-center experience

**DOI:** 10.1186/s40792-016-0183-0

**Published:** 2016-06-03

**Authors:** Go Anegawa, Yuichiro Nakashima, Yoshihiko Fujinaka, Ikuo Takahashi

**Affiliations:** Department of Surgery, Saga-Ken Medical Centre Koseikan, 400 Kase-machi, Nakabaru, Saga 840-8571 Japan; Department of Surgery, Matsuyama Red Cross Hospital, Ehime, Japan

**Keywords:** LADG, Elderly patients, Early gastric cancer

## Abstract

**Background:**

The safety and efficacy of laparoscopy-assisted distal gastrectomy (LADG) for early gastric cancer have been demonstrated in clinical studies. The aim of this study was to clarify the safety and efficacy of LADG in patients ≥80 years of age with early gastric cancer, an American Society of Anesthesiologists (ASA) classification of 1–2, and a performance status (PS) of 0–1.

**Case presentation:**

From April 2009 to July 2011, 12 elderly patients aged ≥80 years and 43 younger patients underwent LADG for early gastric cancer. Seven of the 55 patients underwent LADG and simultaneous surgery including surgery for colorectal cancer, cholecystectomy, or other conditions. Forty-eight of the 55 patients who underwent only LADG were studied. Demographics and postoperative outcomes were compared.

**Results:**

The postoperative complication rate, time to first ambulation, time to first flatus, time to first fluid intake, and postoperative hospital stay were similar in these two groups. Nutritional status as assessed by body weight, serum albumin, and total protein at 1 and 3 months after surgery was also similar in these two groups.

**Conclusions:**

Postoperative outcomes were acceptable in the elderly patients included in the study. LADG for early gastric cancer is a safe and effective treatment in elderly patients aged ≥80 years with an ASA status of 1–2 and PS of 0–1.

## Background

The safety and efficacy of laparoscopic gastrectomy in the treatment of early gastric cancer have been demonstrated in several clinical studies [1–4]. A recent randomized controlled trial showed that quality of life was better with laparoscopic gastrectomy than open gastrectomy [5]. In Japan, the number of laparoscopic gastrectomies is increasing because of a high incidence of gastric cancer [6].

For elderly patients, who have poorer organ function and less capacity to withstand surgical stress, a less invasive laparoscopic approach may be particularly beneficial. Previous reports have shown that laparoscopy-assisted distal gastrectomy (LADG) for early gastric cancer is a safe and effective treatment in elderly patients aged ≥70 years [6, 7]. Although the average human life expectancy is approximately 80 years and 33.3 % of patients with gastric cancer are 70–79 years old in Japan, the safety and efficacy of LADG have not been fully demonstrated in an elderly population aged ≥80 years [8]. Because it is believed that LADG for elderly patients will increase in the future, the safety and efficacy of LADG for elderly patients should be evaluated in detail.

The aim of this study was to assess the safety and efficacy of LADG in elderly patients aged ≥80 years with early gastric cancer, an American Society of Anesthesiologists (ASA) classification of 1–2, and a performance status (PS) of 0–1 before extending the indication for LADG to all elderly patients, including those with an ASA classification of 3–4. Short-term surgical variables and outcomes were retrospectively compared between patients aged ≥80 and ≤79 years.

## Case presentation

In this retrospective study, we reviewed 55 patients who underwent LADG for early gastric cancer between April 2009 and July 2011 at our department. Seven of the 55 patients underwent LADG and simultaneous surgery, including 3 operations for colorectal cancer, 2 cholecystectomies, 1 extirpation of an intra-abdominal tumor, and 1 clipping of the inferior mesenteric artery after endovascular aneurysm repair for an abdominal aortic aneurysm. Forty-eight of the 55 patients were classified into 2 groups based on age. Ten patients (20.8 %) were ≥80 years of age. The clinicopathological features of these groups were reviewed using hospital records and compared with those of 38 younger patients, defined as those ≤79 years of age. In both groups of patients, all tumors were adenocarcinomas invading the mucosa or submucosa of the stomach. The indications for LADG included the following: depth of tumor invasion limited to the mucosa or submucosa, absence of lymph node metastases in preoperative examinations, and any histological type of adenocarcinoma including poorly differentiated adenocarcinoma. Furthermore, the location of the tumor was limited to the middle or lower part of the stomach. The operative risk was assessed according to the ASA classification and PS. The indication for LADG was limited in patients with an ASA of 1 or 2 and PS of 0 or 1. The operations were performed by a single surgeon (G.A.).

The patient management protocols in the perioperative and postoperative periods were similar in the two groups. Drinking and diet were initiated when the first passage of flatus was recognized, and the diets in the two groups were similar. The criterion for discharge was identical in the two groups, namely, that the patients could take in more than 50–60 % of a normal diet without fever, pain, diarrhea, or vomiting.

The following parameters were recorded retrospectively: age, sex, body mass index (BMI), estimated glomerular filtration rate (eGFR), percent vital capacity (%VC), presence of comorbidities, operation time, estimated blood loss, conversion to open surgery, time to first flatus, time to first fluid intake, length of postoperative hospital stay, and postoperative complications. Three months after the operation, patients were interviewed regarding gastrointestinal symptoms such as heartburn, nausea, gastric fullness, diarrhea, and dumping syndrome. Nutritional parameters after the operation were assessed by body weight and laboratory data (serum albumin and total protein).

### Surgical procedures

Laparoscopic resections with dissection of the regional lymph nodes and lymph nodes along the left gastric artery or the common hepatic artery and the celiac axis were performed as follows. The lymph node dissection range followed the classification of the Japanese Research Society for Gastric Cancer [9]. A camera port was introduced into the umbilicus, and two 12-mm trocars were introduced into the left and right lateral quadrants using a flexible fiberscope with a 10-mm tip (Olympus Optical, Ltd., Tokyo, Japan). The gastric arteries were laparoscopically clipped and divided with adequate lymphadenectomy using a five-port technique. The duodenum was mobilized and then staple-transected using a linear stapling device with a disposable GI cartridge (Echelon Flex 60-3.5; Ethicon Endo-Surgery, Cincinnati, OH). A small midline upper abdominal incision, 4 cm in length, was made and retracted using a wound-sealing device (Alexis Wound Retractor; Applied Medical, Rancho Santa Margarita, CA). After extra-abdominal exteriorization of the distal stomach through this mini-laparotomy, the resection line of the stomach was determined by palpation of the marking clips placed under the guidance of preoperative gastroendoscopy. The stomach was divided using a linear stapler along the planned resection line from the greater curvature to the lesser curvature. Furthermore, Roux-en-Y reconstruction was performed through this mini-laparotomy. Abdominal irrigation with 2 L warm saline was performed, and a closed-type silicon drain (J-VAC; Ethicon, Inc., Somerville, NJ) was subsequently placed around the gastrojejunal anastomosis.

### Statistical analysis

All continuous data are presented as the mean ± SD. Statistical analyses were performed using the Student’s *t* test and the *χ*^2^ test. A two-way repeated-measures analysis of variance (ANOVA) was used to compare the nutritional status as assessed by body weight, serum albumin, and total protein at 1 and 3 months after surgery. A value of *P* < 0.05 was regarded as significant.

### Results

A total of 48 LADGs were performed, and no conversion to open surgery was recorded. The clinical backgrounds of the patients are summarized in Table 1. The eGFR was significantly lower and the ASA class was significantly higher in patients aged ≥80 years than in patients ≤79 years. The details of the operative status are given in Table 2. The operation time (311 ± 47 min in the elderly group vs. 324 ± 72 min in the younger group), estimated blood loss (72 ± 97 g in the elderly group vs. 125 ± 167 g in the younger group), and intraoperative complications were not significantly different between the two groups. The details of postoperative recovery in the two groups are given in Table 3. The time to first ambulation was 1.6 ± 0.5 days in the elderly group and 1.5 ± 0.6 days in the younger group. The time to first flatus was 2.8 ± 0.8 days in the elderly group and 2.2 ± 0.8 days in the younger group. The time to first fluid intake was 4.4 ± 1.2 days in the elderly group and 3.7 ± 1.9 days in the younger group. The postoperative hospital stay was 15.8 ± 6.4 days in the elderly group and 15.3 ± 4.4 days in the younger group. These differences were not statistically significant.

**Table 1:** Preoperative characteristics of patients undergoing LADG

	Elder *n* = 10	Younger *n* = 38	*P* value
Age	83 ± 2.8	66 ± 7.2	<0.0001
Gender (male/female)	7/3	28/10	
BMI	21.6 ± 3.8	22.9 ± 3.6	N.S.
eGFR (ml/min/1.73 m^2^)	56.3 ± 13.9	70.6 ± 19.7	0.014
%VC, %	109 ± 20	105 ± 18.1	N.S.
ASA class (1/2)	0/10	13/25	0.028
Performance status (0/1)	8/2	38/0	
Comorbidity			
Hypertension, *n* (%)	2 (20)	6 (15.7)	
Ischemic heart disease, *n* (%)	1 (10)	1 (2.6)	
Arrhythmia, *n* (%)	1 (10)	1 (2.6)	
Diabetes mellitus, *n* (%)	0	3 (7.8)	
Chronic hepatitis C, *n* (%)	1 (10)	1 (2.6)	
NASH, *n* (%)	0	1 (2.6)	
History of renal cancer, *n* (%)	1 (10)	1 (2.6)	
End-stage renal failure, *n* (%)	0	1 (2.6)	
History of cerebral infarction, *n* (%)	0	5 (13.1)	
Dementia, *n* (%)	1 (10)	0	

N.S. means " not significant"

**Table 2:** Operative characteristics

	Elder *n* = 10	Younger *n* = 38	*P* value
Operation time, (min)	311 ± 47	324 ± 72	N.S.
Blood loss, (g)	72 ± 97	125 ± 167	N.S.
Conversion to open surgery	0	0	

N.S. means " not significant"

**Table 3:** Postoperative progress

	Elder *n* = 10	Younger *n* = 38	*P* value
Time to first ambulation, (days)	1.6 ± 0.5	1.5 ± 0.6	N.S.
Time to first flatus, (days)	2.8 ± 0.8	2.2 ± 0.8	N.S.
Time to first fluids, (days)	4.4 ± 1.2	3.7 ± 1.9	N.S.
Hospital stays, (days)	15.8 ± 6.4	15.3 ± 4.4	N.S.

N.S. means " not significant"

The postoperative complications in the two groups are listed in Table 4. The incidence rate of postoperative complications was not significantly different between the two groups. In terms of complications related to reconstruction, neither anastomotic leakage nor stricture was seen in either group. One patient in the elderly group and two in the younger group developed intra-abdominal abscessation, which was treated with antibiotic medication. No operation-related death occurred.

**Table 4:** Postoperative complications

	Elder *n* = 10	Younger *n* = 38	*P* value
Complication (+)	1 (10)	3 (7.8)	N.S.
Leakage	0	0	
Intra-abdominal abscess, *n* (%)	1 (10)	2 (5.2)	N.S.
Ileus, *n* (%)	0	1 (2.6)	N.S.

N.S. means " not significant"

**Fig. 1:**
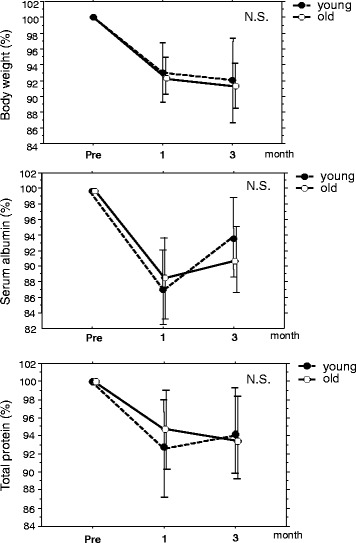
Postoperative nutritional status during the first 3 months after surgery

The postoperative nutritional status during the first 3 months after surgery is shown in Fig. 1. No significant difference was observed between the groups in the rate of weight loss at 1 and 3 months postoperatively. The serum albumin and total protein levels at 1 and 3 months after surgery did not differ between the two groups. Gastrointestinal symptoms were examined 3 months after surgery (Table 5). The incidence of nausea, gastric fullness, diarrhea, and dumping syndrome did not differ between the groups.

**Table 5:** Postoperative gastrointestinal symptoms at 3 months after surgery

Gastrointestinal symptoms	Elder *n* = 10	Younger *n* = 38	*P* value
Heartburn, *n* (%)	0	0	
Nausea, *n* (%)	0	1 (2.6)	N.S.
Gastric fullness, *n* (%)	1 (10)	2 (5.2)	N.S.
Diarrhea, *n* (%)	1 (10)	1 (2.6)	N.S.
Dumping syndrome, *n* (%)	0	1 (2.6)	N.S.

N.S. means " not significant"

### Discussion

The average human life expectancy is increasing in Japan, which already has one of the longest life expectancies in the world. As a result of this trend toward longer lives, the proportion of elderly patients diagnosed with gastric cancer is also increasing [10]. Because many elderly patients have comorbidities [11, 12], surgeons often hesitate to perform operations.

For stage IA–IIIC gastric cancer according to the classification of the Japanese Research Society for Gastric Cancer [9], the standard therapy is undoubtedly curative surgical resection except in early cases, which may warrant endoscopic resection. Considering the increased risk of surgery in elderly patients who present with comorbidities, there has been controversy over whether surgical resection is the best way to care for these patients. However, surgery for gastric cancer has been shown to significantly improve the prognosis of older patients aged ≥85 years [10]. Surgery should not be immediately rejected for patients of very advanced ages. We reviewed the experience of LADG in patients with early gastric cancer and compared the results in patients ≥80 and ≤79 years of age.

Previous studies have shown that laparoscopic gastrectomy is considered to be less invasive than open gastrectomy and that LADG offers particular advantages to elderly patients aged ≥70 years with early gastric cancer. These advantages include rapid return of gastrointestinal function, fewer complications, and a shorter hospital stay [6, 7]. In a previous study of pulmonary function after gastrectomy, the decrease in forced vital capacity after LADG was less than that after open gastrectomy because of a reduction in postoperative pain [13]. The reduced impairment of pulmonary function after LADG compared with open gastrectomy may be especially beneficial in elderly patients.

Although the most frequent concurrent disease in the elderly group was cardiovascular disease in this study, cardiovascular complications did not occur after LADG in either group. A previous report showed that the increase in intra-abdominal pressure during pneumoperitoneum can lead to an increase in systemic vascular resistance and central filling pressures and a decrease in the cardiac index, which may be detrimental in elderly patients with limited cardiac reserve [14]. Indeed, the true effect of pneumoperitoneum on the cardiopulmonary physiology is not completely clear. Further investigation is required, and different methods, such as the use of low-pressure pneumoperitoneum or a gasless laparoscopic approach, may be flexibly used [15, 16]. As in a previous report [12], renal function in patients aged ≥80 years was significantly lower in this study; however, there was no postoperative renal dysfunction in either group. LADG may have little influence on renal function. Although the preoperative ASA class was significantly higher in all patients aged ≥80 years, there was no mortality, and the overall morbidity was similar in both groups. A previous report also showed that for patients with ASA classifications of 3 and 4, laparoscopic colectomy was associated with similar postoperative mortality but less overall morbidity, quicker return of bowel function, and shorter length of hospital stay compared with open colectomy [17]. As far as we investigated, there was no report of LADG for elderly patients with ASA classifications of 3 and 4. Further study is needed to evaluate the safety and efficacy of LADG before extending the indication for LADG to all elderly patients, including those with an ASA classification of 3–4.

## Conclusions

In summary, we reviewed the experience of LADG in elderly patients aged ≥80 years in our institution. Our results showed that LADG is a safe and effective procedure in elderly patients aged ≥80 years selected by an ASA classification of 1–2 and PS of 0–1 in terms of short-term operative results, postoperative complications, postoperative nutritional status, and postoperative gastrointestinal symptoms. LADG poses few limitations for elderly patients with an ASA classification of 1–2 and PS of 0–1.

## Ethics approval and consent to participate

All procedures were in accordance with the ethical standards of the responsible committees on human experimentation (institutional and national) and with the Helsinki Declaration of 1964 and later versions. The patient gave informed consent for the procedures.
